# MiR‐140 modulates the inflammatory responses of *Mycobacterium tuberculosis*‐infected macrophages by targeting TRAF6

**DOI:** 10.1111/jcmm.14472

**Published:** 2019-06-14

**Authors:** Xiaofei Li, Shan Huang, Tingting Yu, Guiliang Liang, Hongwei Liu, Dong Pu, Niancai Peng

**Affiliations:** ^1^ School of Life Science and Technology Xi'an Jiaotong University Xi'an City China; ^2^ Department of Clinical Laboratory The Third People's Hospital of Kunming City Kunming China; ^3^ School of Mechanical Engineering Xi'an Jiaotong University Xi'an City China

**Keywords:** human peripheral blood mononuclear cells, *M tb* survival, miR‐140, *Mycobacterium tuberculosis*, pro‐inflammatory cytokines, TRAF6

## Abstract

This study aimed to examine miR‐140 expression in clinical samples from tuberculosis (TB) patients and to explore the molecular mechanisms of miR‐140 in host‐bacterial interactions during *Mycobacterium tuberculosis* (*M tb*) infections. The miR‐140 expression and relevant mRNA expression were detected by quantitative real‐time PCR (qRT‐PCR); the protein expression levels were analysed by ELISA and western blot; *M tb* survival was measured by colony formation unit assay; potential interactions between miR‐140 and the 3′ untranslated region (UTR) of tumour necrosis factor receptor‐associated factor 6 (TRAF6) was confirmed by luciferase reporter assay. MiR‐140 was up‐regulated in the human peripheral blood mononuclear cells (PBMCs) from TB patients and in THP‐1 and U937 cells with *M tb* infection. Overexpression of miR‐140 promoted *M tb* survival; on the other hand, miR‐140 knockdown attenuated *M tb* survival. The pro‐inflammatory cytokines including interleukin 6, tumour necrosis‐α, interleukin‐1β and interferon‐γ were enhanced by *M tb* infection in THP‐1 and U937 cells. MiR‐140 overexpression reduced these pro‐inflammatory cytokines levels in THP‐1 and U937 cells with *M tb* infection; while knockdown of miR‐140 exerted the opposite actions. TRAF6 was identified to be a downstream target of miR‐140 and was negatively modulated by miR‐140. TRAF6 overexpression increased the pro‐inflammatory cytokines levels and partially restored the suppressive effects of miR‐140 overexpression on pro‐inflammatory cytokines levels in THP‐1 and U937 cells with *M tb* infection. In conclusion, our results implied that miR‐140 promoted *M tb* survival and reduced the pro‐inflammatory cytokines levels in macrophages with *M tb* infection partially via modulating TRAF6 expression.

## 1 INTRODUCTION

Tuberculosis (TB) is a serious infectious disease and is caused by *Mycobacterium tuberculosis* (*M tb*) infection, and TB has imposed great threats to human health worldwide.^1^ According to the epidemic statistics, around 9 million individuals were diagnosed as TB patients, and TB caused around 1 million deaths in 2016.^2^ In China, owing to the spread of TB with drug resistance and the dual infection of TB and human immunodeficiency virus, the cases of TB infection have remarkably increased in recent years.^3^Mechanistic studies have shown that *M tb* is an intracellular pathogen, which can parasitize the host's macrophages,^4^ and the elimination of mycobacteria by macrophages can be achieved through different mechanisms including regulating inflammatory responses, modulating host cell‐death programs and regulating phagosome acidification and maturation.^5^ Unfortunately, *M tb* can still survive and replicate via overcoming the microbicidal mechanisms of macrophages. In this regard, it is necessary for us to explore the molecular mechanisms underlying the immunological role of macrophages in *M tb* infection, which may provide us with new strategy for treatment of TB.

MiRNAs belong to a class of small, non‐coding RNAs with 21‐23 nt and can regulate gene expression at the post‐translational level.^6^ MiRNAs can bind to the complementary sites in the 3′ untranslated region (3′UTR) of the targeting genes and subsequently cause degradation of the transcript or translational repression.^7^ MiRNAs are well‐studied for their diverse functions in the aspect of biological processes including cell proliferation, cell differentiation, cell apoptosis, cell death and so on. Dysregulation of miRNAs is closely related with human diseases including cancer, cardiovascular dysfunctions and neurodegenerative diseases.^8^, ^9^, ^10^ Recently, miRNAs have been shown as important regulators in the TB infection. Ghorpade et al, demonstrated that miR‐155 regulated immune responses to mycobacterial infection via modulation of reprogramming processes of the cells.^11^ Further study by Zheng et al, revealed that in the pulmonary TB, miR‐155 and miR‐132 could serve as important diagnostic biomarkers.^12^ Liu et al, showed that TB induced miR‐582‐5p up‐regulation in TB patients and miR‐582‐5p inhibited monocytes apoptosis by targeting forkhead box protein O1.^13^ In addition, miR‐223 was found to exert positive effect on the recruitment of lung neutrophil, which may be related to TB susceptibility,^14^ and miR‐124 could negatively regulate toll‐like receptor signalling in *M tb*‐infected alveolar macrophages.^15^ Recently, studies from Lin et al, demonstrated that miR‐140 was overexpressed in human macrophages infected with *M tb* using microarray analysis,^16^ suggesting the potential actions of miR‐140 in TB. Unfortunately, the role of miR‐140 in TB has not been examined in detail so far.

In our current investigations, miR‐140 up‐regulation was found in the human peripheral blood mononuclear cells (PBMCs) from patients with TB, and further in vitro functional assays were performed to determine the miR‐140 actions on the *M tb* survival and the pro‐inflammatory cytokine levels in the human macrophages with *M tb* infection. In addition, the underlying molecular mechanisms were also assessed by the in vitro functional assays. The current investigations may imply the novel role of miR‐140 in the host‐bacterial interactions during *M tb* infections.

## 2 MATERIALS AND METHODS

### 2.1 Clinical sample collection

Sixty patients with TB and 60 healthy volunteers were recruited in this study at the Third People's Hospital of Kunming City during 2015‐2017. The diagnostic criteria for TB were TB symptoms by a chest X‐ray alone with positivity for *M tb* culture. The TB patients had no immune deficiency disease, hepatitis B, diabetes or other pulmonary‐related complications. Healthy controls were recruited from volunteers who had a routine physical examination at Third People's Hospital of Kunming City. The peripheral blood were collected into ETDA‐treated tubes from patients when admitted to the hospital for the first time. The PBMCs were isolated from the peripheral blood via density gradient centrifugation by Ficoll separation (GE Healthcare, Chicago, USA). After washing with PBS, the PBMCs were then subjected to further analysis. The study protocol was approved by the Institutional Ethics Committee Board of the Third People's Hospital of Kunming City, and the informed consent was obtained from each volunteer.

### 2.2 Cell lines and culture

Two human macrophage cell lines (THP‐1 and U937) were commercially purchased from the ATCC company (Manassas, VA). The THP‐1 and U937 cells were both cultured in RPMI 1640 medium (Thermo Fisher Scientific, Waltham, MA) with the supplement of 10% foetal bovine serum (FBS; Gibco, Thermo Fisher Scientific), sodium pyruvate (1 mmol/L; Sigma, St. Louis, MO), L‐glutamine (2 mmol/L; Sigma), penicillin (100 U/mL; Sigma) and streptomycin (100 mg/mL; Sigma). THP‐1 and U937 cells were maintained in the humidified conditions with 5% CO_2_ at 37°C. The cells were treated with 100 nmol/L phorbol 12‐myristate 13‐acetate (PMA; Sigma) for overnight and then washed for three times. Following PMA stimulation, cells were rested for 3 days before further in vitro studies.

### 2.3 M tb culture and infection

*M tb* (H37RV; the virulent strain) was commercially obtained from ATCC company (Manassas). The *M tb* was cultured in Middlebrook 7H9 liquid medium containing oleic acid albumin dextrose catalase enrichment (Sigma) at 37°C. The infecting human macrophages with *M tb* were performed based on the previously published protocols.^17^, ^18^ Briefly, 1 mL cultures of H37RV mycobacterial strain was pelleted for 2 minutes followed by re‐suspension in RPMI 1640 medium, and the suspension was then vortexed for 2 minutes and sonicated in a bath sonicator (Thomas Scientific, Swedesboro, USA) for 5 minutes. After sonication, dispersed mycobacterial suspensions were allowed to rest for 5 minutes, and the upper 500 μL was used for infecting macrophages. For the infections, the H37RV and macrophages were cultured onto the 24‐well plates. H37RV with different multiplicity of infections (MOIs) were allowed to infect the macrophages for different durations (3, 6, 12, 24 and 48 hours).

### 2.4 MiRNA oligonucleotides, vectors and oligonucleotides transfection

The miRNA oligonucleotides including the mimic and inhibitor for miR‐140 as well as their corresponding negative controls (NCs, the corresponding miRNAs with the scrambled sequence) were commercially obtained from Ribobio (Guangzhou, China). The pcDNA3.1 vector as well as pcDNA3.1 vector with tumour necrosis factor receptor‐associated factor 6 (TRAF6) overexpression were commercially obtained from BlueGene Company (Shanghai, China). The transfection of miRNAs or vectors into macrophage cell lines was performed with Lipofectamine 2000 reagent (Invitrogen, Carlsbad, USA). At 48 hours following transfection, the transfected macrophages were used for other experimental assays. For the infection studies, cells were infected with *M tb* immediately after transfections and at 48 hours after infection, cells were collected for further experimental assays.

### 2.5 Gene expression as measured by quantitative real‐time PCR

Total RNA isolation from cells was performed with TRIzol reagent (Invitrogen) according to the manufacturer's protocol. Total RNA was reversely transcribed into cDNA using the SuperScript III kit (Invitrogen). For the determination of miR‐140 levels, quantitative real‐time PCR (qRT‐PCR) was performed with a Gentier 96E Real‐time PCR system (Xi'an Tianlong Science&Technology Co. Ltd., Xi'an, China) using TaqMan microRNA assay kit (Thermo Fisher Scientific). For the detection of mRNA expression, qRT‐PCR was performed with a Gentier 96E Real‐time PCR system (Xi'an Tianlong Science&Technology Co. Ltd.) using Fast SYBR Green Master Mix kit (Takara, Dalian, China). U6 and glyceraldehyde 3‐phosphate dehydrogenase were chosen as internal controls for miR‐140 and mRNA expression respectively. MiR‐140 and mRNA expressions were quantified by the comparative threshold cycle method.

### 2.6 M tb survival as determined by colony‐forming unit assay

*Mycobacterium tuberculosis* survival after infecting into THP‐1 or U937 cells was assessed by the colony‐forming unit (CFU) assay. Briefly, the transfected human macrophages were infected by *M tb* at 10 MOI for a duration of 48 hours followed by lysing the infected cells with sterile distilled water. Ten‐fold serial dilutions were used for quantitative culturing with each dilution inoculated on Middlebrook 7H10 agar plates containing 10% oleic acid albumin dextrose catalase enrichment (Sigma). The *M tb* was cultured for 3 weeks at 37°C and the CFUs were determined using the standard protocols.

### 2.7 Protein levels as determined by ELISA assay

The medium for culturing THP‐1 and U937 cells that received different treatments was collected for the ELISA analysis of pro‐inflammatory cytokine levels. The protein levels of interleukin 6 (IL‐6), tumour necrosis‐α (TNF‐α), interleukin‐1β (IL‐1β) and interferon‐γ (IFN‐γ) were analysed using the corresponding ELISA kits (Thermo Fisher Scientific).

### 2.8 Bioinformatics prediction and luciferase reporter assay

The complementary sequence between TRAF6 3′UTR and miR‐140 was predicted by TargetScan tool. The wild‐type fragment of TRAF6 3′UTR with the miR‐140 binding sequence was amplified by PCR and inserted into the pGL3 vector (Promega, Madison, USA). The mutation of TRAF6 3′UTR was performed by mutating the relevant bases in miR‐140 seed region using a site‐directed mutagenesis method (Takara). For the luciferase reporter assay, HEK293T cells were cotransfected with miRNA oligonucleotides and Renilla luciferase pRL‐TK vector, and wild‐type/mutant TRAF6 3′UTR reporter vector. At 48 hours following transfection, luciferase activity was determined using a dual‐luciferase assay system (Promega) according to the manufacturer's protocol.

### 2.9 Western blot analysis

Extraction of protein from cells was performed with radioimmunoprecipitation assay buffer lysis buffer (Sigma), and concentrations of the extracted proteins were measured by a bicinchoninic acid protein assay kit (Beyotime, Beijing, China). The extracted proteins (30 µg for each lane) were separated using 10% SDS‐PAGE followed by transferring onto the polyvinylidene difluoride membranes (Millipore, Bedford, USA). The transferred membrane was then incubated with PBS in Tween 20 containing 5% non‐fat milk at room temperature for 1 hour. The membrane was then incubated with rabbit anti‐human TRAF6 (1:1000 dilution; Santa Cruz Biotechnology, Santa Cruz, USA) and mouse anti‐human β‐actin (1:2000 dilution; Santa Cruz Biotechnology) at 4°C overnight. After incubation with primary antibodies, the membrane was then incubated with relevant secondary antibodies conjugated with horseradish peroxidase (Santa Cruz Biotechnology) at room temperature for 2 hours. The western blot bands on the membrane were detected by the enhanced chemiluminescence reagents (Sigma).

### 2.10 Statistical analysis

All the data analyses were performed in the GraphPad Prism Version 6.0 software (GraphPad Prism Software, La Jolla, USA). All the experimental assays were performed in triplicates. The data were shown as mean ± SD. Student's *t* test or one‐way ANOVA followed by Turkey's post hoc test was used to analyse the statistical significance between different treatment groups. The differences were considered to be statistically significant when *P* < 0.05.

## 3 RESULTS

### 3.1 Up‐regulation of miR‐140 in PBMCs from TB patients and M tb‐infected macrophages

**Figure 1:**
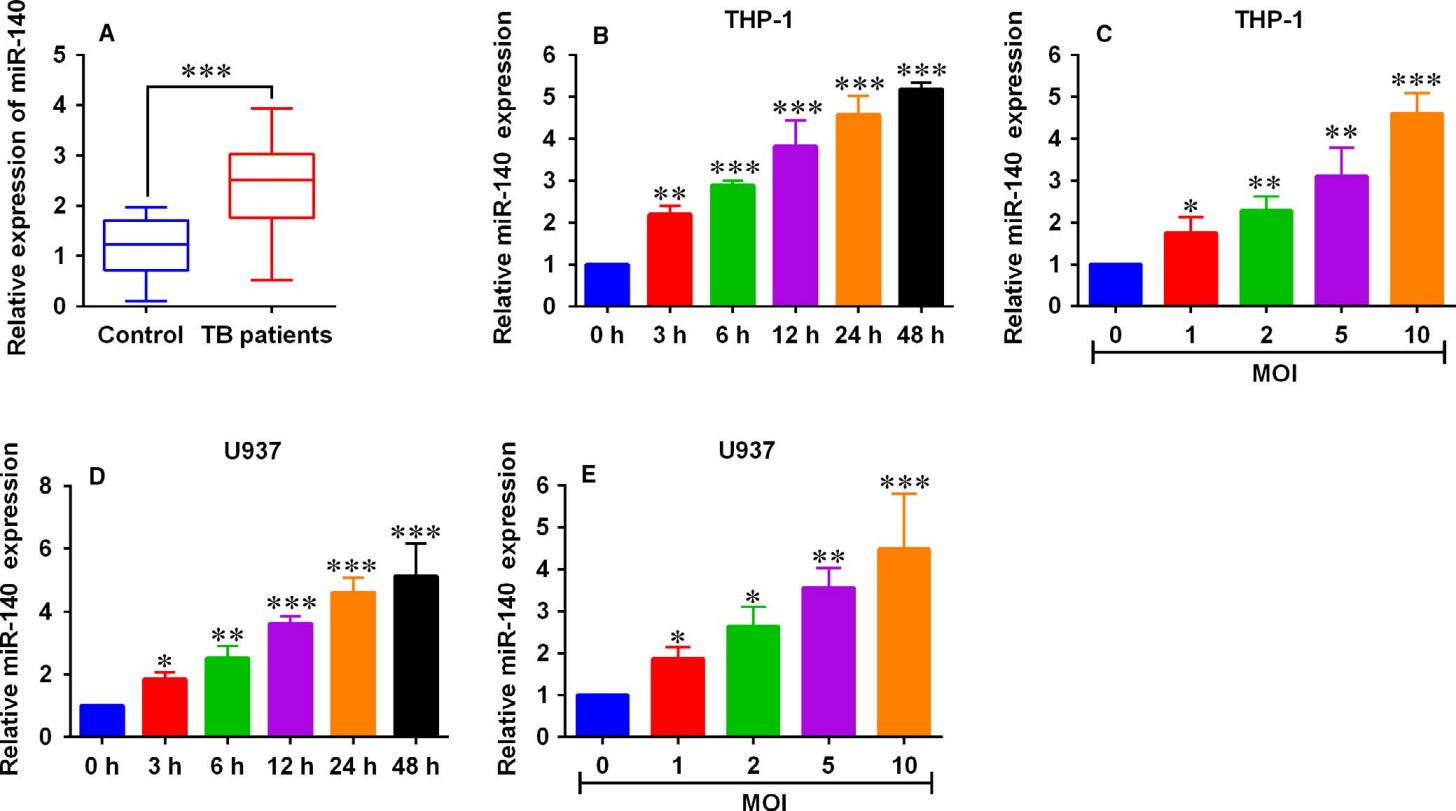
MiR‐140 was up‐regulated in peripheral blood mononuclear cells (PBMCs) from tuberculosis (TB) patients and *Mycobacterium tuberculosis* (*M tb*)‐infected macrophages. A, Quantitative real‐time PCR (qRT‐PCR) analysis of miR‐140 expression levels in PBMCs from normal controls (n = 60) and TB patients (n = 60). B, qRT‐PCR analysis of miR‐140 expression levels in THP‐1 cells that infected with 10 MOI *M tb* for 3, 6, 12, 24 and 48 h respectively. C, qRT‐CPR analysis of miR‐140 expression levels in THP‐1 cells infected with 1, 2, 5 and 10 MOI *M tb*, respectively, for 48 h. D, qRT‐PCR analysis of miR‐140 expression levels in U937 cells that infected with 10 MOI *M tb* for 3, 6, 12, 24 and 48 h respectively. E, qRT‐PCR analysis of miR‐140 expression levels in U937 cells infected with 1, 2, 5 and 10 MOI *M tb*, respectively, for 48 h. *P < 0.05, **P < 0.01 and ***P < 0.001

Firstly, we determined the miR‐140 expression in PBMCs from normal controls and TB patients, and the data revealed the up‐regulation of miR‐140 in the PBMCs from TB patients comparing with healthy controls group (Figure 1A).

To further elucidate the potential role of miR‐140 in TB, we examined the miR‐140 expression levels in the human macrophage cell lines (THP‐1 and U937 cells that have become some of the most widely used in vitro models to study the host and pathogen interaction during *M tb* infection 19) after being infected by *M tb* at different MOIs for different time durations. The results demonstrated that *M tb* infection significantly up‐regulated the miR‐140 expression in both THP‐1 and U937 cells in time‐ and concentration‐dependent manners (Figure 1B‐1E). In the sequent in vitro studies, *M tb* at 10 MOI infecting the macrophages for a duration of 48 hours was used.

### 3.2 MiR‐140 promoted M tb survival in macrophages

**Figure 2:**
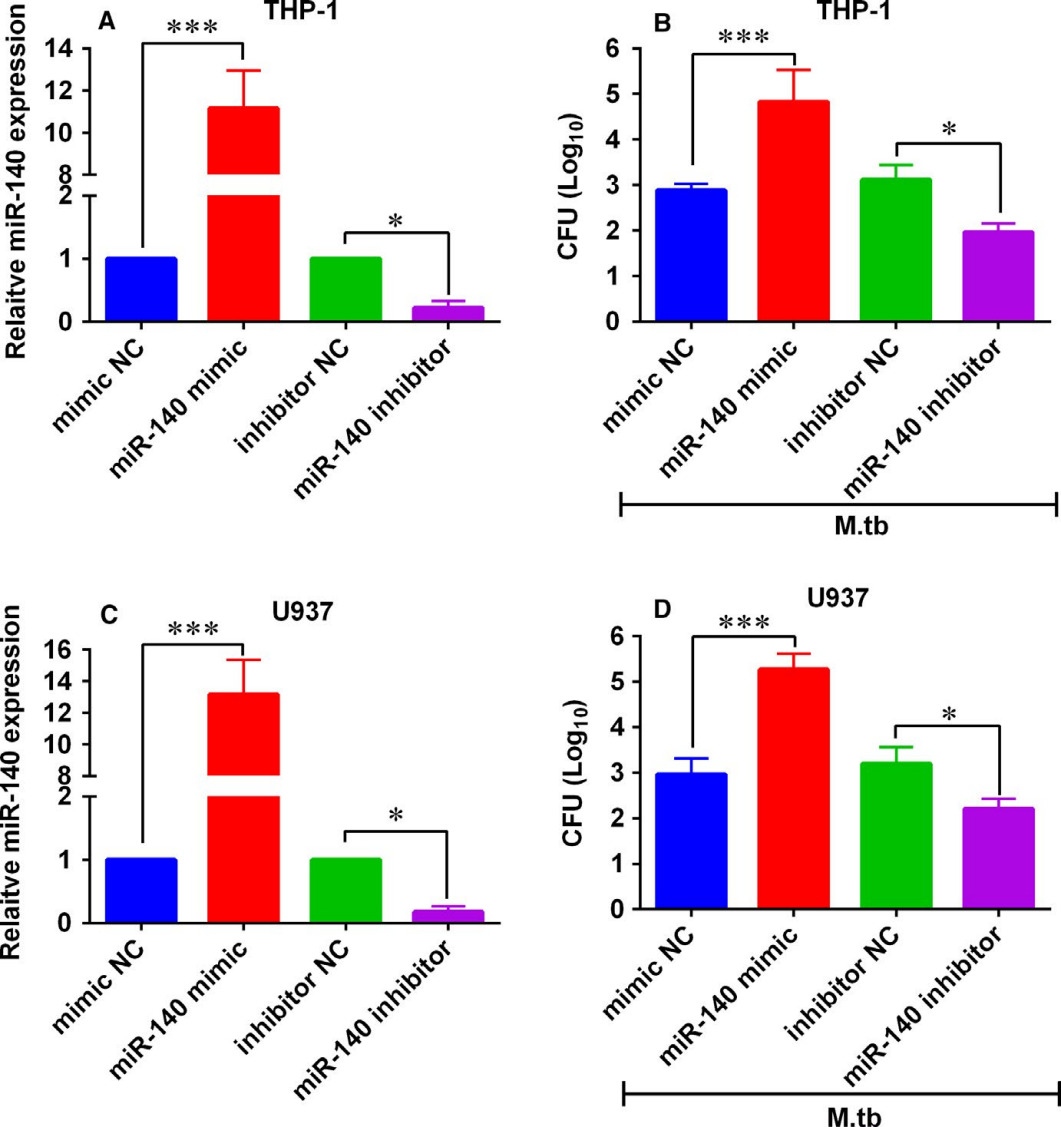
Effects of miR‐140 on the *Mycobacterium tuberculosis* (*M tb*) survival in macrophages. A, Quantitative real‐time PCR (qRT‐PCR) analysis of miR‐140 expression levels in THP‐1 cells after being transfected with miR‐140 mimic, miR‐140 inhibitor and their corresponding negative controls (mimic NC and inhibitor NC). B, The transfected THP‐1 cells were infected with *M tb* and colony‐forming unit (CFU) assay measured the mycobacterial viability. C, qRT‐PCR analysis of miR‐140 expression levels in U937 cells transfected with miR‐140 mimic, miR‐140 inhibitor and their corresponding negative controls (mimic NC and inhibitor NC). D, The transfected U937 cells were infected with *M tb* and CFU assay measured the mycobacterial viability. N = 3. *P < 0.05 and ***P < 0.001

In order to further determine the molecular mechanisms of miR‐140 in TB, we manipulated the miR‐140 expression levels by transfecting human macrophage cell lines with different miRNA oligonucleotides. As shown in Figure 2A, miR‐140 mimic transfection significantly enhanced the miR‐140 expression in THP‐1 cells comparing with mimic NC group; on the other hand, miR‐140 inhibitor transfection suppressed miR‐140 expression in THP‐1 cells comparing with inhibitor NC group. The effects of miR‐140 on mycobacterial viability of *M tb* were evaluated by CFU assay, overexpression of miR‐140 increased the mycobacterial viability and down‐regulation of miR‐140 suppressed the mycobacterial viability (Figure 2B). Consistently, overexpression of miR‐140 increased the mycobacterial viability in *M tb*‐infected U937 cells, and down‐regulation of miR‐140 suppressed the mycobacterial viability in *M tb*‐infected U937 cells (Figure 2C,D).

### 3.3 Effects of M tb infection on the pro‐inflammatory cytokines levels in THP‐1 and U937 cells

**Figure 3:**
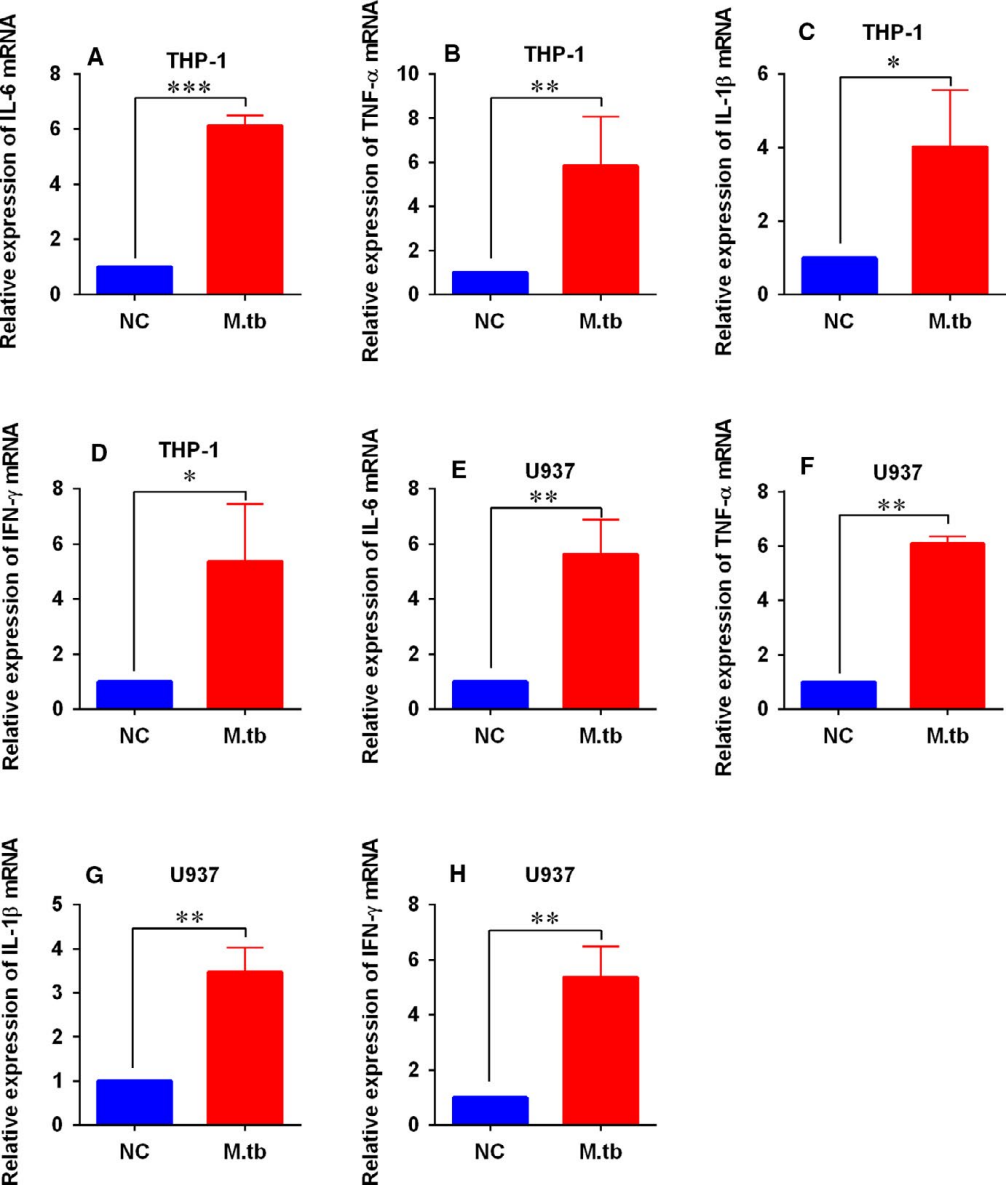
Effects of *Mycobacterium tuberculosis* (*M tb*) infection on the pro‐inflammatory cytokines mRNA expression levels in macrophages. (A‐D) Quantitative real‐time PCR (qRT‐PCR) analysis of interleukin 6 (IL‐6), tumour necrosis‐α (TNF‐α), interleukin‐1β (IL‐1β) and interferon‐γ (IFN‐γ) mRNA expression levels in THP‐1 cells infected with 10 MOI *M tb* for 48 h. (E‐H) qRT‐PCR analysis of IL‐6, TNF‐α, IL‐1β and IFN‐γ mRNA expression levels in U937 cells infected with 10 MOI *M tb* for 48 h. N = 3. *P < 0.05, **P < 0.01 and ***P < 0.001

Firstly, we performed qRT‐PCR to determine the expression of IL‐6, TNF‐α, IL‐1β and IFN‐γ mRNA in the macrophages after being infected by *M tb* (10 MOI). Infecting THP‐1 cells for 48 hours significantly increased the mRNA expression of IL‐6, TNF‐α, IL‐1β and IFN‐γ (Figure 3A‐D). Consistently, infection of *M tb* enhanced the mRNA expression of these pro‐inflammatory cytokines in U937 cells (Figure 3E‐H). Moreover, ELISA assay was performed to determine the protein levels of these pro‐inflammatory cytokines, and *M tb* infection significantly caused the increase in protein levels of these pro‐inflammatory cytokines in both THP‐1 and U937 cells (Table 1).

**Table 1:** Effects of *Mycobacterium tuberculosis* (*M tb*) infection on the pro‐inflammatory cytokines levels in macrophages

Cell lines	Cytokines	Treatments	
		NC	*M tb*
THP‐1	IL‐6 (pg/mL)	48.9 ± 8.5	143.2 ± 12.3***
	TNF‐α (pg/mL)	91.2 ± 22.3	456.4 ± 34.7***
	IL‐1β (pg/mL)	147.6 ± 22.1	402.7 ± 33.9***
	IFN‐γ (pg/mL)	192.5 ± 23.5	489.8 ± 45.5***
U937	IL‐6 (pg/mL)	56.9 ± 11.3	155.2 ± 18.9**
	TNF‐α (pg/mL)	87.3 ± 26.5	412.9 ± 51.2**
	IL‐1β (pg/mL)	119.8 ± 16.7	508.2 ± 51.4***
	IFN‐γ (pg/mL)	222.9 ± 32.6	433.3 ± 67.8**

Abbreviation: IFN‐γ, interferon‐γ; IL‐6, interleukin 6; IL‐1β, interleukin‐1β; TNF‐α, tumour necrosis‐α; NC, negative control.

** *P* < 0.01;

*** *P* < 0.001 relative to NC group.

### 3.4 Effects of miR‐140 on the pro‐inflammatory cytokines expression levels in M tb‐infected THP‐1 and U937 cells

**Figure 4:**
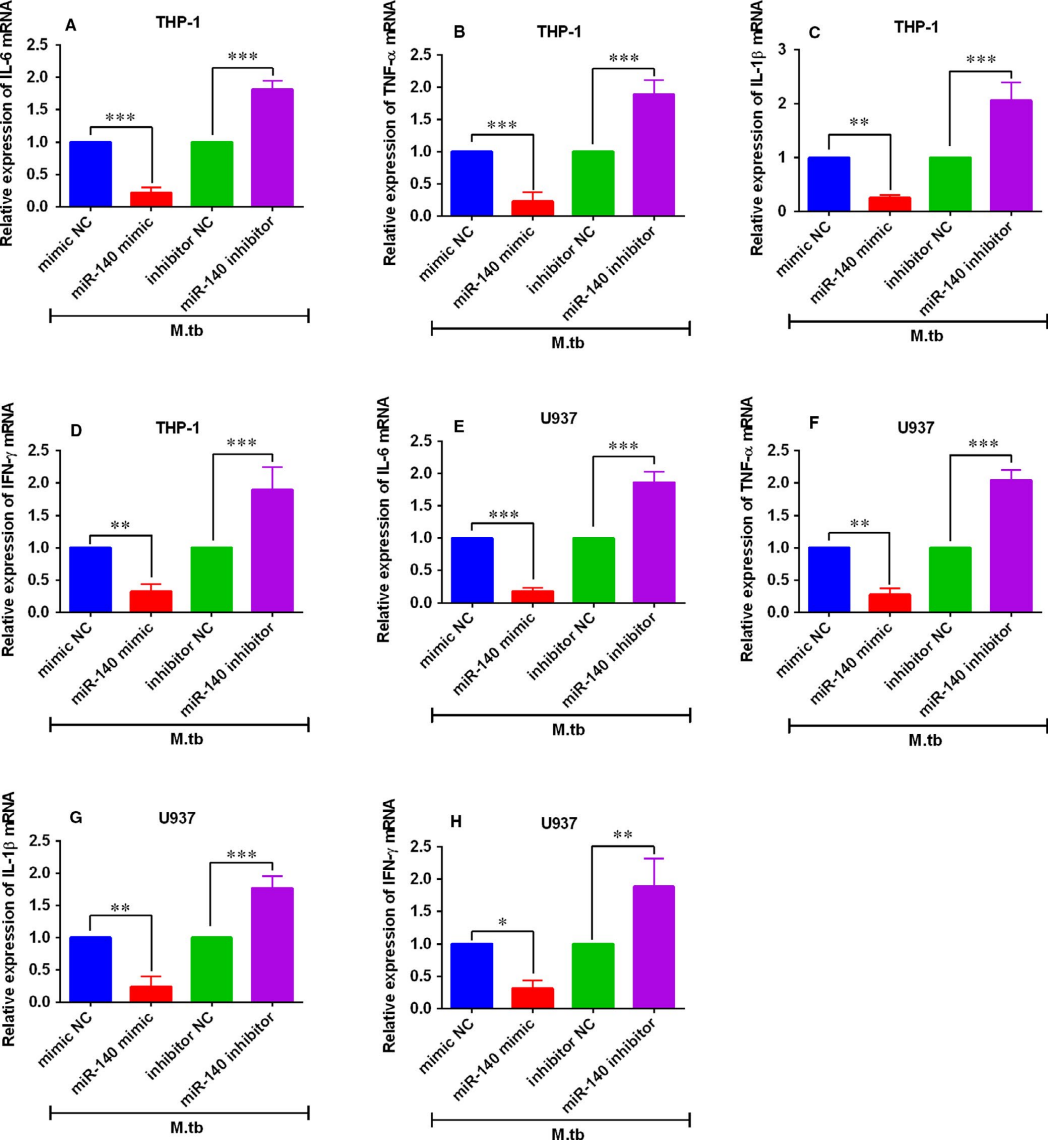
Effects of miR‐140 on the pro‐inflammatory cytokines mRNA expression levels in *M tb*‐infected macrophages. (A‐D) Quantitative real‐time PCR (qRT‐PCR) analysis of interleukin 6 (IL‐6), tumour necrosis‐α (TNF‐α), interleukin‐1β (IL‐1β) and interferon‐γ (IFN‐γ) mRNA expression levels in miRNA oligonucleotides‐transfected THP‐1 cells after *M tb* infection. (E‐H) qRT‐PCR analysis of IL‐6, TNF‐α, IL‐1β and IFN‐γ mRNA expression levels in miRNA oligonucleotides‐transfected U937 cells after *M tb* infection. N = 3. *P < 0.05, **P < 0.01 and ***P < 0.001

For the determination of the roles of miR‐140 in the pro‐inflammatory cytokines release, we first transfected the THP‐1 and U937 cells with different miRNAs, and immediately following the transfections, cells were infected with *M tb* at 10 MOI for 48 hours. MiR‐140 mimic transfection significantly down‐regulated the mRNA expression of IL‐6, TNF‐α, IL‐1β and IFN‐γ in *M tb*‐infected THP‐1 cells compared to mimic NC group; while miR‐140 inhibitor transfection enhanced the expression of the corresponding genes in *M tb*‐infected THP‐1 cells compared to inhibitor NC group (Figure 4A‐D). Similarly, overexpression of miR‐140 also down‐regulated these mRNA in U937 cells and knockdown of miR‐140 had the opposite action in U937 cells (Figure 4E‐H). The ELISA assay results consistently showed that miR‐140 overexpression reduced the protein levels of IL‐6, TNF‐α, IL‐1β and IFN‐γ in *M tb*‐infected THP1 and U937 cells; on the other hand, knockdown of miR‐140 enhanced IL‐6, TNF‐α, IL‐1β and IFN‐γ protein expression in *M tb*‐infected THP‐1 and U937 cells (Table 2).

**Table 2:** Effects of miR‐140 on the pro‐inflammatory cytokines levels in *Mycobacterium tuberculosis* (*M tb*)‐infected macrophages

Cell lines	Cytokines	Treatments			
		mimic NC	miR‐140 mimic	inhibitor NC	miR‐140 inhibitor
THP‐1	IL‐6 (pg/mL)	133.8 ± 12.3	52.7 ± 27.8**	141.9 ± 19.8	179.9 ± 20.1^#^
	TNF‐α (pg/mL)	422.7 ± 39.9	112.6 ± 26.9***	419.8 ± 29.1	511.7 ± 37.8***
	IL‐1β (pg/mL)	422.8 ± 44.2	129.8 ± 24.9***	403.3 ± 31.3	501.1 ± 54.3^#^
	IFN‐γ (pg/mL)	466.9 ± 33.2	212.7 ± 20.7***	430.2 ± 31.6	499.9 ± 32.9^#^
U937	IL‐6 (pg/mL)	172.6 ± 23.9	54.3 ± 22.8***	183.7 ± 25.1	236.6 ± 20.7^#^
	TNF‐α (pg/mL)	436.8 ± 22.3	108.9 ± 27.9***	451.2 ± 21.9	506.7 ± 25.6^#^
	IL‐1β (pg/mL)	529.7 ± 54.9	134.9 ± 33.8***	481.9 ± 34.2	569.7 ± 21.7^#^
	IFN‐γ (pg/mL)	408.8 ± 59.9	293.4 ± 37.6*	379.3 ± 41.2	497.3 ± 26.6^#^

Abbreviation: IFN‐γ, interferon‐γ; IL‐6, interleukin 6; IL‐1β, interleukin‐1β; TNF‐α, tumour necrosis‐α; NC, negative control.

* *P* < 0.05,

** *P* < 0.01,

*** *P* < 0.001 relative to mimic NC group;

^#^ *P* < 0.05,

^##^ *P* < 0.01 relative to inhibitor NC group.

### 3.5 MiR‐140 suppressed TRAF6 expression via targeting the 3’UTR

**Figure 5:**
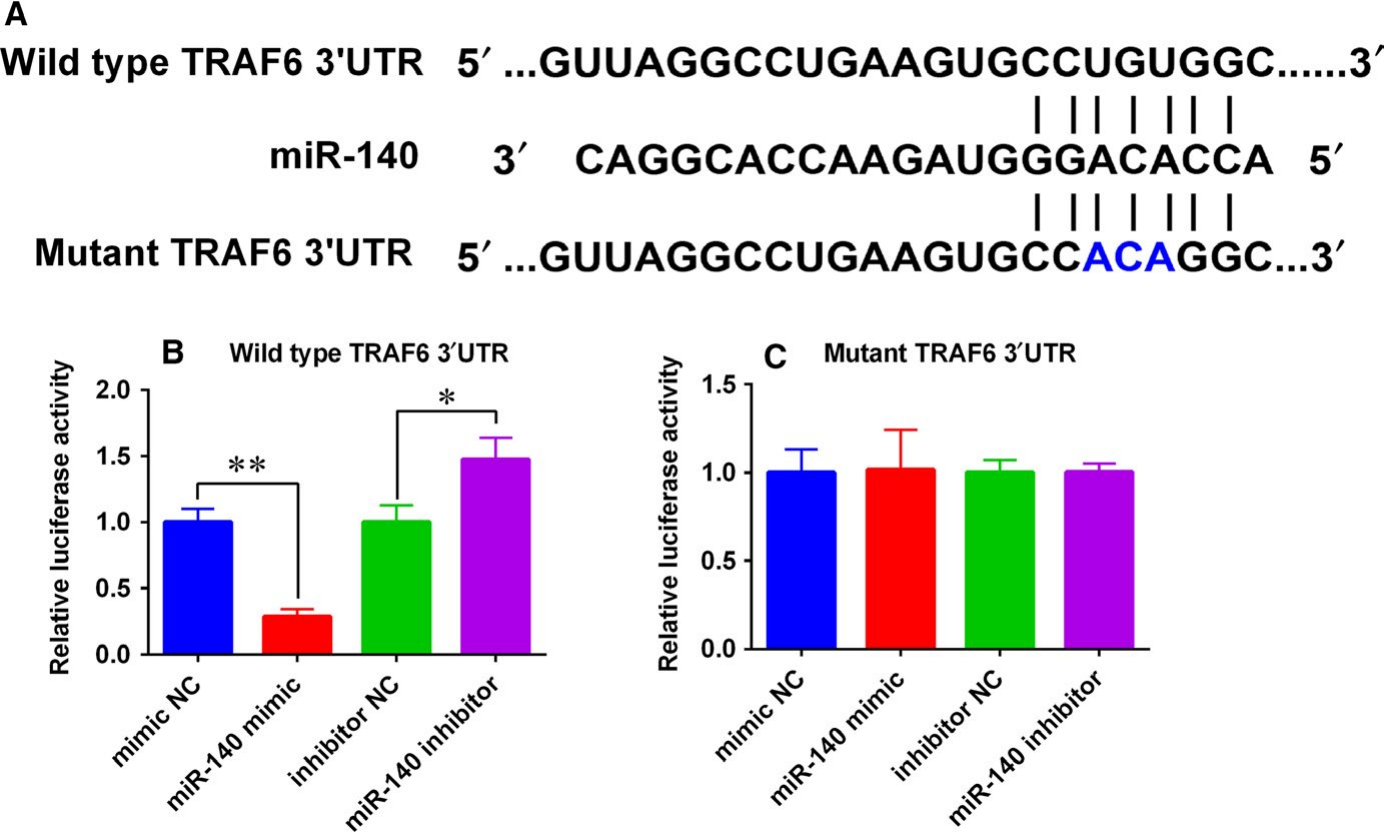
MiR‐140 targets the 3′ untranslated region (UTR) of TRAF6. A, The wild‐type 3′UTR of TRAF6 mRNA contains a putative miR‐140 binding site, and the mutant sequence was indicated with blue letters. B, Relative luciferase activity in HEK293T cells cotransfected with miRNA oligonucleotides (mimic NC, miR‐140 mimic, inhibitor NC and miR‐140 inhibitor) and wild‐type TRAF6 3′UTR‐luciferase reporter vector. C, Relative luciferase activity in HEK293T cells cotransfected with miRNA oligonucleotides (mimic NC, miR‐140 mimic, inhibitor NC and miR‐140 inhibitor) and mutant TRAF6 3′UTR‐luciferase reporter vector. N = 3. *P < 0.05 and **P < 0.01

To further explore the mechanisms of miR‐140 in regulating the inflammatory responses, we performed bioinformatics analysis using Targetscan (www.targetscan.org) prediction software. There are many potential targets were predicted, and TRAF6 was selected from these predicted targets for further validation, as TRAF6 has been reported for its role in *M tb* infection.^20^The bioinformatics analysis showed that TRAF6 was one of the miR‐140‐targeted genes, and the complementary sequences between miR‐140 and TRAF6 3′UTR were shown in Figure 5A. Furthermore, the wild‐type and mutant TRAF6 3′UTR with miR‐140 putative binding sites were amplified by PCR and inserted into the corresponding reporter vector (Figure 5A). MiR‐140 overexpression suppressed the luciferase activity of wild‐type TRAF6 3′UTR‐luciferase reporter vector in HEK293T cells; while knockdown of miR‐140 caused an increase in the luciferase activity of wild‐type TRAF6 3′UTR‐luciferase reporter in HEK293T cells (Figure 5B). Neither miR‐140 overexpression nor miR‐140 knockdown affected the luciferase activity of mutant TRAF6 3′UTR‐luciferase reporter vector in HEK293T cells (Figure 5C).

### 3.6 Effects of TRAF6 overexpression on miR‐140‐mediated pro‐inflammatory cytokines expression in M tb‐infected THP‐1 and U937 cells

**Figure 6:**
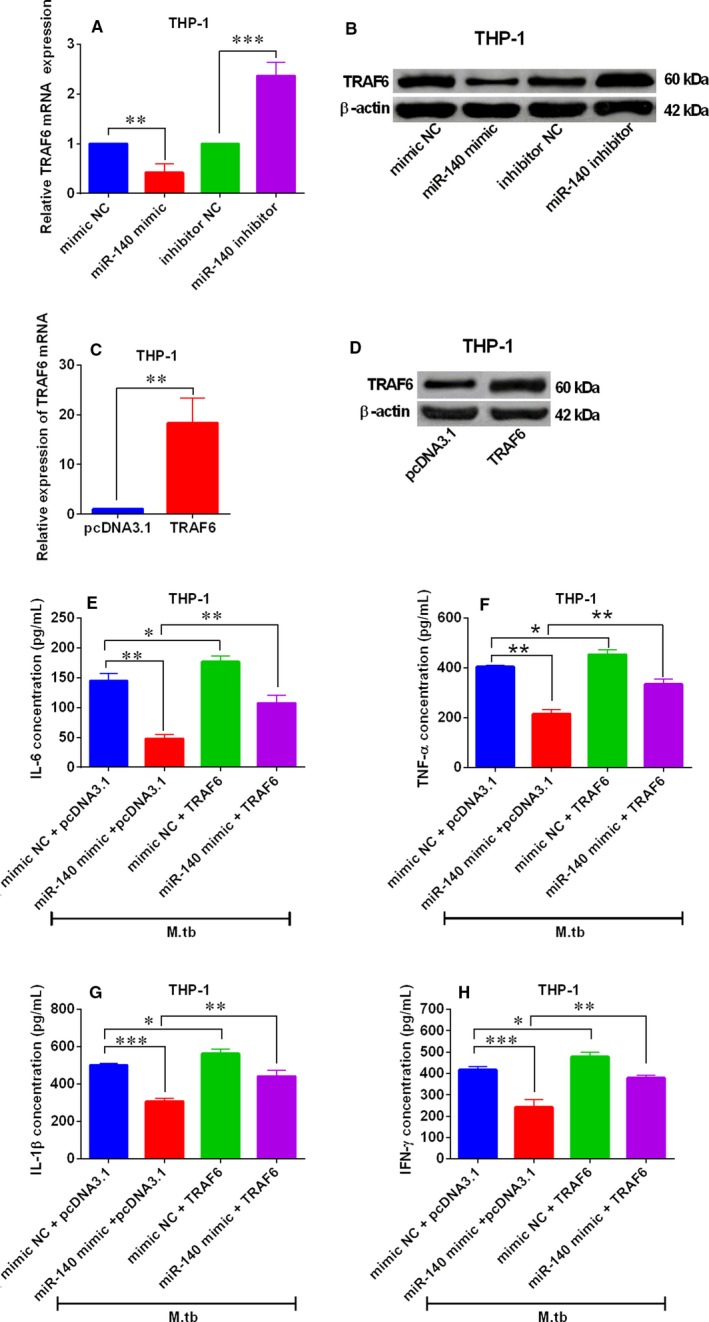
Effects of TRAF6 overexpression on miR‐140‐mediated pro‐inflammatory cytokines expression in *Mycobacterium tuberculosis* (*M tb*)‐infected THP‐1 cells. (A,B) Quantitative real‐time PCR (qRT‐PCR) and western blot analysis of TRAF6 mRNA and protein levels in THP‐1 cells after being transfected with miR‐140 mimic, miR‐140 inhibitor and their corresponding negative controls (mimic NC and inhibitor NC). (C,D) qRT‐PCR and western blot analysis of TRAF6 mRNA and protein levels in THP‐1 cells after being transfected with pcDNA3.1 or pcDNA3.1‐TRAF6. (E‐H) ELISA analysis of interleukin 6 (IL‐6), tumour necrosis‐α (TNF‐α), interleukin‐1β (IL‐1β) and interferon‐γ (IFN‐γ) protein levels with in *M tb*‐infected THP‐1 cells with the pre‐transfections of mimic NC + pcDNA3.1, miR‐140 mimic + pcDNA3.1, mimic NC + pcDNA3.1‐TRAF6 or miR‐140 mimic + pcDNA3.1‐TRAF6. N = 3. *P < 0.05, **P < 0.01 and ***P < 0.001

**Figure 7:**
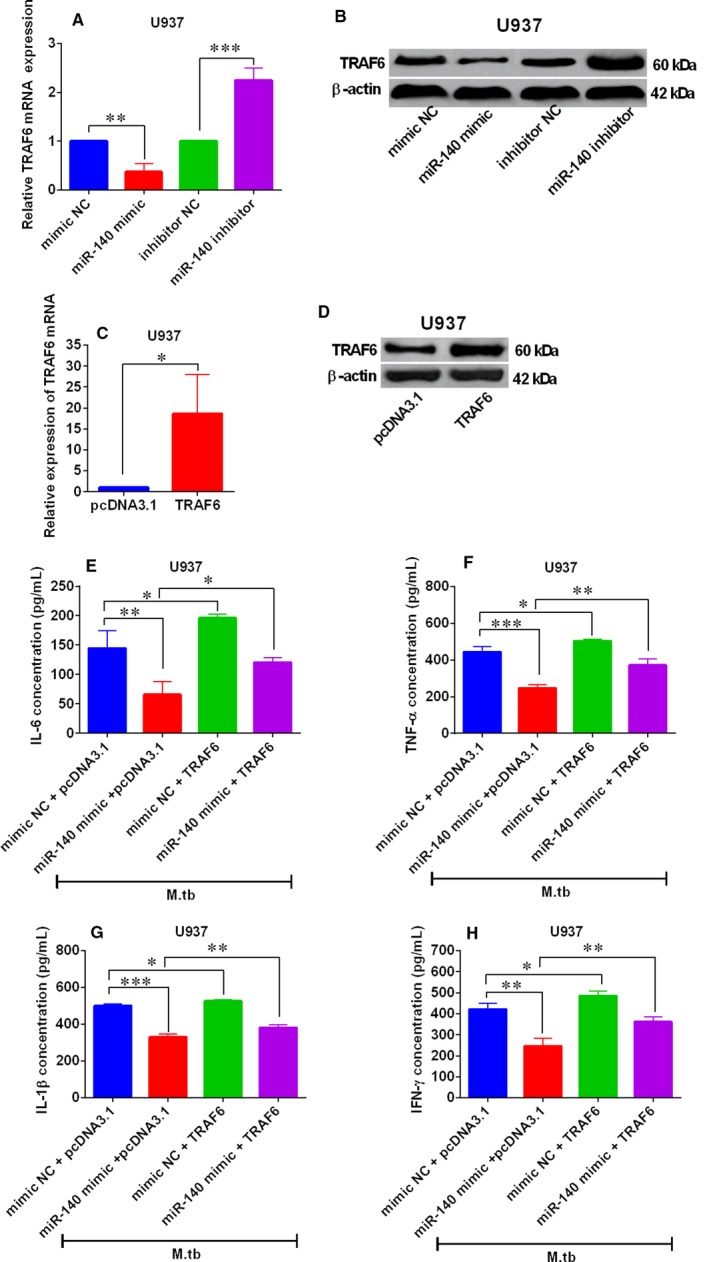
Effects of TRAF6 overexpression on miR‐140‐mediated pro‐inflammatory cytokines expression in *M tb*‐infected U937 cells. (A,B) Quantitative real‐time PCR (qRT‐PCR) and Western blot analysis of TRAF6 mRNA and protein levels in U937 cells after being transfected with miR‐140 mimic, miR‐140 inhibitor and their corresponding negative controls (mimic NC and inhibitor NC). (C,D) qRT‐PCR and western blot analysis of TRAF6 mRNA and protein levels in U937 cells after being transfected with pcDNA3.1 or pcDNA3.1‐TRAF6. (E‐H) ELISA analysis of interleukin 6 (IL‐6), tumour necrosis‐α (TNF‐α), interleukin‐1β (IL‐1β) and interferon‐γ (IFN‐γ) protein levels with in *M tb*‐infected U937 cells with the pre‐transfections of mimic NC + pcDNA3.1, miR‐140 mimic + pcDNA3.1, mimic NC + pcDNA3.1‐TRAF6 or miR‐140 mimic + pcDNA3.1‐TRAF6. N = 3. *P < 0.05, **P < 0.01 and ***P < 0.001

For the determination of the interactions between miR‐140 and TRAF6 in the *M tb*‐infected THP‐1 and U937 cells, we examined the effects of miR‐140 on the TRAF6 mRNA and protein expression levels in the THP‐1 and U937 cells. As shown in Figure 6A and 6, miR‐140 mimic transfection markedly reduced the TRAF6 mRNA and protein levels in THP‐1 cells; while miR‐140 inhibitor transfection caused an increase in TRAF6 mRNA and protein levels in THP‐1 cells (Figure 6A,B), suggesting that miR‐140 negatively regulated the expression of TRAF6 in THP‐1 cells. In addition, TRAF6 overexpression in THP‐1 cells was achieved through transfecting the THP‐1 cells with TRAF6‐overexpressing vector, that is, pcDNA3.1‐TRAF6 (Figure 6C,D). The ELISA results showed that overexpression of TRAF6 increased the protein levels of IL‐6, TNF‐α, IL‐1β and IFN‐γ in THP‐1 cells infected *M tb* (Figure 6E‐H), and also attenuated the suppressive effects of miR‐140 overexpression on these pro‐inflammatory cytokines protein levels in *M tb*‐infected THP‐1 cells (Figure 6E‐H). In agreement with the findings in THP‐1 cells, miR‐140 also negatively regulated the expression of TRAF6 in *M tb*‐infected U937 cells (Figure 7A,B). In addition, overexpression of TRAF6 (Figure 7C,D) significantly increased the protein levels of IL‐6, TNF‐α, IL‐1β and IFN‐γ, and attenuated the suppressive actions of miR‐140 on these pro‐inflammatory cytokines in U937 cells with *M tb* infection (Figure 7E,H).

## 4 DISCUSSION

A growing evidence has implicated the importance of miRNAs for its functional role in TB infection. In the present investigation, we first revealed that miR‐140 was overexpressed in the human PBMCs from the TB patients. Further in vitro functional assays demonstrated that the up‐regulation of miR‐140 was observed in THP‐1 and U937 cells after *M tb* infection, and miR‐140 increased the *M tb* survival and suppressed the expression of pro‐inflammatory cytokines in THP‐1 and U937 cells with *M tb* infection. Further bioinformatics and luciferase assay showed that miR‐140 binds to the 3′UTR of TRAF6 and miR‐140 could negatively affect TRAF6 expression in THP‐1 and U937 cells. Moreover, overexpression of TRAF6 partially reversed the inhibitory actions of miR‐140 overexpression on pro‐inflammatory cytokines in THP‐1 and U937 cells. All in all, these results advanced our knowledge into the novel role of miR‐140 in the host‐bacterial interactions during *M tb* infections.

To our best knowledge, the functional roles of miR‐140 are well‐deciphered in the aspect of human cancers. For instances, knockdown of miR‐140 promoted cancer stem cell self‐renewal in breast cancer via aldehyde dehydrogenase 1/SRY‐box 9 signalling pathway 21; miR‐140 was shown to act as a tumour suppressor in hepatocellular carcinoma and served as an important biomarker for the prognosis of hepatocellular carcinoma.^22^; Duru et al found that nuclear factor, erythroid 2‐related factor 2/miR‐140 signalling, contributed to the radioprotection to human lung fibroblasts 23; in addition, miR‐140 also exerted its tumour‐suppressive actions on gastric carcinoma through targeting YES proto‐oncogene 1.^24^ In terms of infectious diseases, miR‐140 was up‐regulated in the human macrophages upon Leishmania major infection.^25^ MiR‐140 was also found to be up‐regulated in mouse macrophages in responses to lipopolysaccharide stimulation.^26^ Consistently, our present data revealed that up‐regulation of miR‐140 was detected in the human PBMCs isolated from TB patients, and miR‐140 was also up‐regulated in human macrophage cell lines upon *M tb* infection. Further functional assays revealed that miR‐140 exerted suppressive effects on pro‐inflammatory cytokine levels in macrophages after being infected with *M tb*, and the suppressive effects of miR‐140 may be associated with findings that miR‐140 promoted the *M tb* survival within the infected macrophages. All in all, our data may imply that miR‐140 suppressed the pro‐inflammatory cytokine levels in macrophages after being infected with *M tb*, which may contribute to the enhanced effects of miR‐140 on the survival of *M tb*.

To further reveal the mechanistic actions of miR‐140‐mediated host‐bacterial interactions during *M tb* infections, we used the TargetScan tool to do the bioinformatics analysis, and TRAF6 was identified to be one of the targets of miR‐140 and selected for further investigation for its documented role in *M tb* infection.^20^, ^27^ Furthermore, miR‐140 could negatively regulate the expression of TRAF6 in human macrophages. TRAF6 belongs to the TRAF protein family and plays important roles in mediating the signal transduction from members of the tumour necrosis factor receptor superfamily. TRAF6 can relay on MyD88‐dependent toll‐like receptors signalling, which resulted in nuclear factor kappa‐light‐chainenhancer of activated B cells (NF‐κB)/mitogen‐activated protein kinase signalling pathway activation, and ultimately inducing inflammatory cytokines production.^28^ Studies from Niu et al have shown that miR‐125a suppressed cytokines production in macrophages and promotes *M tb* survival via attenuating the activity of NF‐κB signalling through suppressing TRAF6 expression.^20^ Similarly, our present data showed that TRAF6 overexpression caused an increase in the levels of pro‐inflammatory cytokines in macrophages after being infected with *M tb*. More importantly, TRAF6 overexpression partially reversed the inhibitory actions of miR‐140 overexpression on the expression of pro‐inflammatory cytokines in macrophages after being infected with *M tb*. All in all, these data showed that miR‐140 modulated immune responses of macrophages to *M tb* infection partially via targeting TRAF6.

There are several limitations in the current investigations. Firstly, the miR‐140 expression was only investigated in the isolated PBMCs from TB patients, and the determination of miR‐140 expression levels in the sputum from TB patients may provide more solid evidence for the role of miR‐140 in TB. Secondly, because of the difficulties in accessing the clinical features of the TB patients, whether miR‐140 correlated with the clinical features of the TB patients and whether its expression in PBMCs was returned to the levels similar to the healthy volunteers should be determined in the further studies. Thirdly, our study mainly focused on the in vitro functional assays, and future studies may employ in vivo studies to confirm the role of miR‐140 in TB, and performing mice infection experiments followed by quantification of miR‐140 levels and pro‐inflammatory cytokines would be more supportive for our current findings. Fourthly, the downstream targets of miR‐140 were not limited to TRAF6, and it is necessary for us to further explore other interaction between miR‐140 and other targets in the future studies. In addition, future work to confirm the findings in primary human monocyte‐derived macrophages will be warranted.

## 5 CONCLUSIONS

In conclusion, we demonstrated that up‐regulation of miR‐140 was detected in the human PBMCs from the TB patient, and the up‐regulation of miR‐140 was also found in the human macrophages upon *M tb* infection. Further in vitro mechanistic studies indicated that miR‐140 promoted *M tb* survival and suppressed pro‐inflammatory cytokines production in *M tb*‐infected macrophages partially via modulating TRAF6 expression.

## CONFLICT OF INTEREST

None.

## AUTHOR CONTRIBUTIONS

XL and NP conceived and designed the study. XL and SH performed the experiments and wrote the manuscript. TY, GL and HL collected clinical samples and analysed the data. DP performed the statistical analysis. All authors read and approved the final manuscript.

## Data Availability

All the data generated in the study are available upon reasonable request.
